# Implementation of Surgical Safety Checklists in Orthopaedic Surgery: A Narrative Review of Compliance, Barriers, and Future Improvements

**DOI:** 10.7759/cureus.99257

**Published:** 2025-12-15

**Authors:** Zubair Younis, Muhammad A Hamid, Karim Rezk, Jay Shah

**Affiliations:** 1 Orthopaedics, The Royal Wolverhampton NHS Trust, Wolverhampton, GBR; 2 Orthopaedic Surgery, University Hospitals Birmingham, Birmingham, GBR; 3 Trauma and Orthopaedics, Airedale NHS Foundation Trust, Keighley, GBR; 4 General Surgery, The Royal Wolverhampton NHS Trust, Wolverhampton, GBR

**Keywords:** compliance, human factors, implementation barriers, orthopaedic surgery, patient safety, surgical safety checklist

## Abstract

The introduction of the World Health Organization’s (WHO) Surgical Safety Checklist (SSC) marked a turning point in global surgical safety, standardising perioperative processes and promoting teamwork across disciplines. While the SSC has demonstrated significant reductions in surgical morbidity and mortality, its implementation in orthopaedic surgery remains uniquely challenging due to procedural complexity, implant verification demands, and frequent laterality checks. Despite high reported compliance, persistent errors such as wrong-site surgery, equipment malfunction, and implant mismatch underscore a disconnect between checklist completion and true surgical readiness.

This narrative review examines the evolution, current use, and effectiveness of the SSC within orthopaedic surgery, highlighting barriers such as hierarchical theatre culture, workflow pressures, and logistical challenges that often reduce engagement to a “tick-box” exercise. Facilitators, including leadership involvement, multidisciplinary communication, simulation-based training, and continuous audit feedback mechanisms, have been identified as key enablers of sustained improvement.

Emerging innovations such as digital checklist integration, real-time data auditing, and artificial intelligence-assisted verification present new opportunities to enhance reliability, accountability, and adaptability in orthopaedic theatres. However, achieving meaningful and lasting impact requires a paradigm shift from procedural adherence to behavioural fidelity and cultural transformation. The review concludes by outlining a future research agenda focused on orthopaedic-specific checklist tailoring, contextual implementation science, and development of standardised outcome metrics. Ultimately, the goal is to evolve the SSC from a procedural formality into an adaptive safety framework that reflects the complexity and precision of modern orthopaedic surgery.

## Introduction and background

Efforts to improve surgical safety have evolved considerably since the World Health Organization (WHO) introduced the Surgical Safety Checklist (SSC) in 2008 as part of the Safe Surgery Saves Lives campaign [1]. The checklist was designed to standardise perioperative practices and enhance teamwork through structured communication among theatre personnel. Subsequent evidence has consistently demonstrated that its use reduces surgical morbidity and mortality across diverse healthcare systems [2]. 

Orthopaedic surgery, however, presents particular challenges for the implementation of safety checklists. Procedures often require extensive instrumentation, complex implant verification, and confirmation of laterality, factors that heighten the risk of adverse events such as wrong-site surgery and retained foreign objects [3]. Despite significant technological advances, orthopaedics remains among the surgical specialities most frequently associated with “never events”. Between 2012 and 2020, orthopaedic operations accounted for approximately one-third of all such events reported to the National Health Service (NHS) England, most commonly involving wrong implants or incorrect operative sites [4]. 

Empirical studies show that compliance with safety checklists does not always translate into error prevention. Thomasson et al., in a prospective quality improvement study, observed that 33% of orthopaedic cases still encountered intraoperative equipment or implant deficiencies even when SSCs were fully completed [5]. These failures, ranging from missing or non-sterile equipment to instrument malfunction, caused avoidable delays and highlighted a gap between checklist documentation and genuine situational readiness. Their findings suggest that while SSCs are valuable, their standalone use is insufficient in high-complexity settings like orthopaedics, where system coordination and communication failures are common. 

Recent systematic reviews confirm that the checklist’s success depends more on how it is implemented than on the checklist itself. Alsadoun et al. reported that variation in outcomes across studies was largely attributable to differences in local adaptation, staff engagement, and organisational culture [6]. Similarly, Mohamed et al. identified hierarchical barriers, workflow interruptions, and inadequate leadership support as recurring obstacles to effective checklist use in orthopaedic theatres [7]. These challenges often lead to “tick-box” compliance rather than meaningful safety verification. 

At the same time, integration of multidisciplinary safety systems such as the Surgical Patient Safety System (SURPASS) has shown promise in further reducing postoperative complications, reoperations, and readmissions [8]. Speciality-specific initiatives, including the American Academy of Orthopaedic Surgeons’ (AAOS) “Sign Your Site” campaign, have also demonstrated that targeted verification processes can significantly decrease wrong-site and implant-related errors. 

This narrative review examines the current landscape of SSC use within orthopaedic surgery, focusing on compliance patterns, common barriers, and areas for future development. It also considers how emerging strategies such as digital checklist integration, real-time auditing, and artificial intelligence (AI)-supported verification may enhance reliability and efficiency. 

## Review

Methodology

This narrative review was based on a focused literature search of key medical databases, including PubMed, Medical Literature Analysis and Retrieval System Online (MEDLINE), Google Scholar, and Scopus, targeting high-impact clinical trials, cohort studies, audits, quality improvement studies, and existing reviews examining the World Health Organization SSC and surgical safety systems, particularly in orthopaedic practice. Articles were selected based on relevance, methodological quality, and citation impact, with emphasis on landmark trials, implementation studies, and orthopaedic-specific safety evidence.

Historical context and rationale

The modern emphasis on surgical safety emerged in response to growing awareness of preventable medical errors within healthcare systems. The Institute of Medicine’s landmark report To Err is Human estimated that medical errors caused between 44,000 and 98,000 deaths annually in the United States, positioning healthcare errors as a major public health concern [4]. This report served as a catalyst for global patient safety movements, inspiring systematic reforms aimed at minimising human error through standardisation, communication, and cross-disciplinary accountability [7].

The concept of using structured checklists to enhance safety originated in high-reliability industries such as aviation, where pre- and post-flight checklists dramatically reduced catastrophic failures. Translating this model into healthcare, early surgical checklists were primarily speciality-specific, such as antibiotic prophylaxis checklists or thromboembolism prevention protocols, but lacked a comprehensive framework encompassing the entire perioperative pathway [7]. Recognising this gap, the WHO launched the Safe Surgery Saves Lives initiative in 2007, which resulted in the development of the 19-item SSC [1]. This tool was designed to improve consistency in patient identification, surgical site verification, anaesthetic safety, and postoperative planning across all surgical specialities.

Early multicentre studies demonstrated the SSC’s ability to reduce postoperative complications and mortality. The pivotal WHO trial involving 7,688 patients across eight hospitals globally reported a reduction in complications from 11% to 7% and mortality from 1.5% to 0.8% following checklist adoption [1]. Subsequent trials confirmed similar benefits across varied clinical settings, reinforcing the SSC as a cornerstone of modern surgical safety culture [2]. The checklist’s three-phase structure, Sign In, Time Out, and Sign Out, was intentionally designed to mirror aviation protocols, promoting situational awareness and encouraging shared responsibility among team members [7].

Orthopaedic surgery has historically been at the forefront of surgical safety reforms due to its procedural complexity and high incidence of wrong-site operations. Before the WHO initiative, orthopaedic-specific safety programs such as the American Academy of Orthopaedic Surgeons’ Sign Your Site campaign and the North American Spine Society’s Sign, Mark, and X-ray protocol were introduced to minimise laterality and procedural errors [9]. These initiatives laid the groundwork for integrating the SSC into orthopaedic practice, where verification of laterality, implant type, and equipment readiness is essential.

Despite widespread adoption, implementation of the SSC in orthopaedic surgery remains inconsistent. Studies show that compliance alone does not guarantee safety; meaningful engagement, leadership involvement, and adaptation to local workflows are critical determinants of success [5,6]. Orthopaedic procedures often require multiple instrument sets, specialised implants, and radiological guidance, all of which increase logistical complexity and the potential for intraoperative equipment failures. Thomasson et al. observed that even with checklist completion, 33% of orthopaedic procedures in their institution experienced missing or malfunctioning instruments, highlighting that checklist adherence without systemic coordination is insufficient to ensure surgical readiness [5]. Table 1 provides a summary of all the components of SSC.

**Table 1:** SSC checklist summary Reference: [1]

Phase	Key Components & Verification Items	Primary Objective & Timing
Sign In	Conducted before the induction of anaesthesia with the patient conscious. • Patient confirmation: Name, Procedure, Site marked. • Allergies? • Anaesthetic safety: Airway plan, Equipment & drugs, Blood availability discussed. • Stop before you block (specific check to prevent wrong-sided nerve blocks).	To confirm patient identity, procedure, and site; ensure anaesthetic safety and equipment readiness; and address critical pre-induction risks.
Time Out	Conducted immediately prior to knife-to-skin, with the entire team present. • Introductions (Team members). • Confirm patient’s: Name, Procedure, Site and side, Imaging displayed. • Allergies (second check). • Surgeon: Significant blood loss? • Team check (tasks completed): Diathermy on, Antibiotics given, Anaesthetic plan, Warming on, VTE prophylaxis on. • Final verification: 'Anyone not happy to start?'	To perform a final verification, ensure all critical tasks are completed, foster team communication, and align on the plan before incision.
Sign Out	Conducted before any team member leaves the operating room. • Procedure recorded as... • Counts correct (instruments, sponges, needles). • Specimens labelled. • Packs removed/labelled. • Lines flushed. • Equipment problems identified. • Post-op plan: Specific concerns? VTE & antibiotic plan. Day case?	To ensure procedural accuracy, correct counts, proper specimen handling, address equipment issues, and communicate the recovery plan.

The rationale for this review lies in addressing these persisting gaps between checklist completion and effective implementation. By critically analysing compliance patterns, barriers, and emerging innovations such as digital checklist integration and real-time data monitoring, this study aims to provide an updated understanding of how SSCs can be optimised for orthopaedic practice. The ultimate goal is to foster a safety culture that extends beyond procedural verification toward continuous, team-based situational awareness, ensuring that the SSC not only standardises processes but also transforms them into meaningful patient safety outcomes.

Current compliance and barriers in orthopaedic practice

The global uptake of the WHO's SSC has been remarkable since its introduction, yet compliance and consistency in its execution remain highly variable across surgical specialities. Within orthopaedics, reported adherence rates range widely between 40% and 98%, reflecting substantial differences in institutional protocols, team engagement, and local safety cultures [6]. While most centres have adopted the checklist in principle, its practical application often fails to achieve the intended depth of multidisciplinary collaboration and verification.

Studies examining SSC compliance in orthopaedics reveal that completion rates alone do not guarantee effective safety practice. Sewell et al. found that despite a dramatic increase in checklist completion from 7.9% to 96.9% after educational interventions, the associated reduction in postoperative complications was modest and statistically nonsignificant [10]. This discrepancy underscores that procedural completion without active engagement does little to improve outcomes. Similarly, Thomasson et al. reported that even when the SSC was fully completed before surgery, 33% of orthopaedic procedures at their institution experienced intraoperative equipment failures or implant-related deficiencies, leading to significant time delays and workflow disruptions [5]. These findings suggest that in orthopaedic practice, checklist compliance must be paired with a culture of accountability and continuous team communication to ensure meaningful impact.

A growing body of evidence identifies several recurring barriers that hinder consistent SSC use. Hierarchical dynamics within the operating theatre are among the most significant obstacles. Surgeons and anaesthetists may resist nurse-led checklist facilitation, perceiving it as a challenge to established authority structures [7]. This lack of psychological safety can suppress open dialogue, particularly during critical verification stages such as Time Out and Sign Out. The literature also highlights workflow-related barriers, including the perception that checklists prolong surgical preparation or interrupt concentration during busy or emergency sessions [11]. In high-turnover orthopaedic theatres, where efficiency pressures are pronounced, teams may perform the checklist hastily or omit certain steps altogether to maintain schedule adherence.

Logistical and environmental factors further complicate SSC adherence in orthopaedics. The speciality’s reliance on large volumes of implants and specialised instrumentation increases the risk of missing, contaminated, or malfunctioning equipment. In such settings, even minor coordination lapses between surgical, nursing, and sterile processing teams can result in case delays or compromised sterility, undermining patient safety despite nominal checklist completion. The frequent involvement of implant representatives and multiple scrub teams introduces additional communication challenges, emphasising the need for structured interprofessional briefings before incision.

Institutional support and training also play critical roles in sustaining high compliance. Alsadoun et al. found that hospitals with dedicated safety champions and routine audit-feedback mechanisms demonstrated higher fidelity in SSC implementation compared with centres lacking such oversight [6]. Conversely, inconsistent leadership engagement or lack of follow-up monitoring led to checklist fatigue and declining adherence over time. Education and simulation-based training have proven effective in improving team understanding of checklist rationale, yet these efforts require ongoing reinforcement to counter complacency and staff turnover [8].

Despite these barriers, evidence indicates that well-implemented SSC programmes can significantly improve safety outcomes in orthopaedic surgery. Enhanced multidisciplinary participation, clear role definition, and incorporation of postoperative debriefs have been associated with reductions in communication errors, wrong-site surgeries, and retained foreign objects [10]. Sustained improvement, however, demands institutional investment in training, regular auditing, and adaptation of checklists to the specific procedural and equipment demands of orthopaedic practice.

Overall, compliance in orthopaedics reflects a broader challenge: transitioning from checklist completion to checklist integration within everyday theatre culture. While awareness and adoption are nearly universal, variability in engagement, leadership, and workflow design continues to limit the full realisation of the SSC’s potential. Addressing these issues requires not only technical adherence but also a transformation in communication culture, one that prioritises shared accountability, open dialogue, and continuous reflection across all members of the surgical team (Table 2).

**Table 2:** Key findings from pivotal clinical studies WHO: World Health Organization; SSC: Surgical Safety Checklist

Study	Setting/Sample	Focus	Key Findings	Impact on Outcomes
Mohamed et al., 2025 [7]	Orthopaedic literature review	Best practices for SSC implementation in orthopaedics	Highlighted the need for customised orthopaedic checklists, staff training, and continuous audit	Supported speciality-specific adaptation of SSC to maximise safety benefits in orthopaedic surgery
Storesund et al., 2020 [8]	Multispecialty surgical population (including orthopaedics)	Combined perioperative safety system with WHO SSC	Combined safety system reduced complications, reoperations, and readmissions compared to SSC alone	Demonstrated the added benefit of integrating SSC with broader perioperative safety pathways
Fudickar et al., 2012 [11]	Multispecialty surgical units, Germany	Effect of WHO SSC on complications and communication	Significant reduction in postoperative complications and improved team communication following SSC implementation	Reinforced SSC as an effective tool for improving safety outcomes and intraoperative teamwork

Facilitators and enablers of successful checklist use

The successful implementation of SSCs in orthopaedics is highly dependent on institutional, interpersonal, and procedural facilitators that strengthen compliance and teamwork. Leadership engagement stands as one of the most critical enablers. Studies have demonstrated that visible and consistent support from surgical and departmental leadership enhances staff motivation, ensures accountability, and reinforces checklist adherence as part of organisational culture [2]. Strong leadership commitment transforms checklist completion from a routine task into a shared professional responsibility [1].

Effective communication and teamwork are repeatedly identified as the backbone of checklist success. Russ et al. found that structured checklist discussions significantly improved interdisciplinary communication and reduced hierarchical barriers, particularly between surgeons, anaesthetists, and nursing teams [12]. Similarly, Catchpole et al. emphasised that task coordination and psychological safety are vital for error detection, allowing team members to voice concerns without fear of reprisal, a key behavioural determinant of checklist effectiveness [13].

Educational initiatives and simulation-based training further enable robust implementation. Sewell et al. demonstrated that compliance rates increased from 7.9% to 96.9% following structured education, underscoring that checklist adoption must be accompanied by hands-on, multidisciplinary training [10]. Continuous auditing and feedback loops, as advocated by Cima et al., provide iterative reinforcement, helping teams identify lapses and sustain improvement over time [14].

Technological integration is an emerging enabler. Digital checklist platforms, real-time audit tools, and electronic verification systems reduce human error and enhance data accuracy, especially in complex orthopaedic workflows [7]. Furthermore, aligning checklist execution with human-factor engineering principles such as workflow design and ergonomic cues enhances reliability and minimises disruption [15].

Finally, cultivating a positive safety culture underpins all successful implementation efforts. Recent studies have highlighted that routine briefings, debriefings, and acknowledgement of checklist successes foster collective ownership of safety outcomes [4,2]. When team members perceive the checklist as a genuine enabler of patient safety rather than an administrative obligation, adherence improves, and the likelihood of preventable complications decreases dramatically.

Impact on clinical outcomes

The introduction of the WHO SSC has been consistently associated with improved surgical outcomes, but the magnitude of benefit depends heavily on implementation quality and contextual adaptation. Early multicentre trials demonstrated significant improvements in both morbidity and mortality when the SSC was correctly used. In the landmark study by Haynes et al. involving 7,688 patients across eight hospitals worldwide, complication rates fell from 11% to 7%, and perioperative mortality decreased from 1.5% to 0.8% [1]. These findings were later reinforced by van Klei et al., who showed a 36% relative reduction in in-hospital mortality following checklist introduction in a multicentre Dutch cohort, and de Vries et al., whose SURPASS trial demonstrated a 47% decline in complications and a 62% reduction in mortality when a structured checklist system was implemented throughout the perioperative pathway [16,17].

Subsequent systematic reviews and meta-analyses confirmed these findings across diverse surgical specialities and settings. Bergs et al. and Treadwell et al. both reported statistically significant decreases in overall postoperative morbidity, surgical-site infection, and unplanned reoperations, though they emphasised heterogeneity in study design and implementation fidelity [18,19]. More recent high-quality studies, such as Haugen et al. and Storesund et al., demonstrated that the degree of checklist compliance and team engagement was directly proportional to reductions in surgical complications, reoperations, and readmissions [2,8].

Within orthopaedics, checklist implementation has yielded speciality-specific benefits. Sewell et al. reported a reduction in communication errors and near-miss events after checklist adoption in trauma and orthopaedic theatres, while Vohra et al. demonstrated a 40% decrease in wrong-site surgery incidents after the introduction of a modified orthopaedic SSC integrated with the “Sign Your Site” campaign [10,20]. Similarly, the Canadian Orthopaedic Association’s “Operate Through Your Initials” initiative and the AAOS “Sign Your Site” programme contributed to marked reductions in wrong-site and implant-related errors by standardising pre-incision verification [9]. The key findings and impacts on clinical outcomes from pivotal studies are summarised in Table 3.

**Table 3:** Key findings from pivotal clinical studies WHO: World Health Organization; SSC: Surgical Safety Checklist

Study	Setting/Sample	Focus	Key Findings	Impact on Outcomes
Sewell et al., 2011 [10]	UK trauma & orthopaedic theatres	Evaluated the WHO SSC use in orthopaedic patients	Checklist compliance increased from 7.9% to 96.9% after structured education and implementation	Improved team communication and reduced process errors; demonstrated training as a key enabler
Thomasson et al., 2016 [5]	Orthopaedic theatres (implants and equipment preparation)	Assessed whether SSC prevented implant/equipment errors	Despite checklist completion, implant/equipment problems persisted, indicating systemic gaps	Highlighted the need for orthopaedic-specific checklist items and improved logistical integration
Vohra et al., 2016 [20]	Multicentre orthopaedic evaluation	Impact of SSCs and “Sign Your Site” campaigns on wrong-site surgery	Wrong-site events reduced by ~40% after structured site-marking and SSC reinforcement	Demonstrated value of combining SSCs with specialty-specific verification pathways

Checklist use has also improved perioperative process indicators that mediate clinical outcomes. Neily et al., analysing 108 Veterans Health Administration hospitals, found a 14% reduction in mortality after implementing a comprehensive checklist-based team training programme [21]. Adherence to timely antibiotic prophylaxis, temperature regulation, and deep-vein thrombosis prophylaxis has improved significantly in checklist-enabled environments [15]. In orthopaedics, structured Sign In and Time Out steps have improved implant verification accuracy and reduced intraoperative delays related to missing instruments or implant mismatch [5].

Despite these gains, inconsistent or superficial implementation can blunt the clinical effect. Fourcade et al. and Russ et al. identified “tick-box” behaviour and time pressure as contributors to diminished impact [15,22]. Studies that measured outcomes alongside compliance levels found that hospitals with poor engagement derived minimal benefit [2]. Conversely, centres that fostered open communication and multidisciplinary participation saw sustained reductions in morbidity and mortality.

Beyond traditional outcome metrics, SSC use has shown positive effects on culture, efficiency, and cost. Treadwell et al. estimated that complication reduction through checklist use could save £15-20 million annually in the NHS [19]. Enhanced team communication and reduced operative delays have translated into shorter theatre turnover times and improved staff satisfaction [22].

Collectively, these data demonstrate that SSCs substantially improve both clinical and process outcomes when implemented with fidelity, training, and local adaptation. In orthopaedic surgery, where laterality errors, implant complexity, and equipment logistics pose distinct risks, checklists deliver the greatest impact when integrated with discipline-specific safety initiatives and supported by a culture of shared accountability.

Future directions and innovations

As healthcare systems evolve toward digital integration, multidisciplinary collaboration, and precision-driven quality improvement, the next phase of SSC development will depend on technology-enhanced workflows, behavioural reinforcement, and system-wide cultural change. While traditional paper-based SSCs have proven their worth in improving communication and reducing errors, their future success in orthopaedic surgery will hinge on adaptability, data integration, and sustained engagement.

Digitalisation and Real-Time Data Integration

The transition from paper to digital checklists represents one of the most promising developments in surgical safety. Electronic SSCs, integrated into electronic health records (EHRs), can automate prompts, ensure real-time compliance monitoring, and reduce omission errors. Studies have shown that digital systems enhance accuracy, enable timestamp verification, and allow post-procedure audits without interrupting workflow [6,8]. Additionally, cloud-based dashboards enable near-instant feedback and analytics, allowing institutions to track adherence trends and identify recurrent failure points [22]. In orthopaedics, integration of implant tracking systems and barcoding technologies with checklist platforms can ensure correct implant selection and traceability, reducing costly never events related to the wrong prosthesis or side.

AI and Predictive Safety Tools

Emerging research explores AI and machine learning as adjuncts to checklist-based safety systems. AI-assisted verification tools can cross-check consent forms, imaging data, and surgical planning software to flag inconsistencies before incision [7]. Predictive algorithms that analyse workflow deviations, instrument scanning data, or physiological parameters can warn surgical teams of potential complications or protocol breaches in real time. Haugen et al. proposed that these tools could provide early detection of non-compliance events, thereby enhancing situational awareness in complex orthopaedic operations [2]. However, widespread integration will require rigorous validation, robust cybersecurity, and training to ensure usability across multidisciplinary teams.

Speciality-Specific Checklist Adaptation

The orthopaedic environment demands customisation beyond the general WHO SSC structure. Future iterations of checklists should embed orthopaedic-specific verification items such as implant size, laterality, power tool readiness, fluoroscopy setup, and sterile field fluid labelling [5]. Speciality organisations like the AAOS have already pioneered context-specific tools such as Sign Your Site and the Surgical Site Confirmation Protocol, but these require modernisation to integrate seamlessly with digital platforms [9]. Evidence suggests that tailored, contextually relevant checklists improve compliance and team engagement far more effectively than generic templates.

Embedding Human Factors and Cognitive Engineering

Human-factor engineering principles are increasingly recognised as essential for sustaining checklist performance. Fourcade et al. emphasised that cognitive load, time pressure, and hierarchical dynamics are among the most persistent barriers to meaningful checklist use [15]. Future SSC design should therefore focus on cognitive ergonomics, optimising checklist length, sequencing, and visual design to fit naturally into surgical routines. Simulation-based implementation and iterative human-factor testing can ensure usability and minimise resistance from clinical staff. Adopting “pause points” aligned with procedural flow, as recommended by Russ et al., may reinforce compliance while reducing perceived interruptions [22].

Team Training, Leadership, and Culture Reinforcement

Sustained improvement in outcomes requires institutional commitment to training and leadership reinforcement. Building on evidence, future strategies should prioritise multidisciplinary team training, simulation-based communication exercises, and regular debriefings focused on error recognition and learning [21]. Embedding SSC metrics within performance evaluations and continuing professional development frameworks can further institutionalise the checklist as a safety habit rather than an administrative duty. Leadership walk-rounds, peer-review audits, and safety champions can maintain momentum and model best practice within orthopaedic theatres [7].

Global Harmonisation and Cross-Speciality Learning

Despite its global reach, SSC implementation remains inconsistent between health systems. Future research should explore cross-speciality harmonisation to share lessons between orthopaedics and other high-risk surgical fields. International registries and multicentre databases could aggregate real-world outcome data to refine checklist components dynamically. The GlobalSurg Collaborative and WHO-led initiatives already provide frameworks for multi-country benchmarking; applying similar models to orthopaedic safety checklists could accelerate evidence-driven improvement [2,6].

Measuring Meaningful Compliance

A shift from measuring checklist completion to assessing “meaningful compliance” is essential for future evaluation. Fourcade et al. and Russ et al. argue that true adherence involves active team engagement, dialogue, and cross-verification, not just tick-box confirmation [15,22]. Advanced auditing systems combining digital logs with behavioural observation could quantify real engagement levels, linking them to clinical outcomes and guiding targeted interventions.

Knowledge gaps and research agenda

Although the WHO SSC has become an essential component of perioperative safety worldwide, critical gaps remain in the understanding of its long-term effectiveness, speciality-specific adaptation, and sustainability within orthopaedic surgery. Studies consistently highlight that while compliance rates appear high, meaningful engagement and measurable outcome improvement are inconsistent, reflecting deeper systemic and behavioural challenges [5,6].

From Compliance Metrics to Behavioural Fidelity

A recurring gap across SSC literature is the overreliance on checklist completion as a surrogate for success. Studies demonstrate that superficial or perfunctory completion rarely translates into improved outcomes [15,22]. Behavioural fidelity, defined as authentic team engagement, verbal confirmation, and shared situational awareness, remains underexplored. Future research should employ ethnographic observation, audio-visual analysis, or real-time digital monitoring to assess communication quality during checklist use [23]. These approaches would clarify the behavioural mechanisms by which the SSC influences safety outcomes.

Orthopaedic-Specific Validation and Tailoring

Current evidence is dominated by general surgical data, leaving orthopaedic subspecialties underrepresented. Thomasson et al. observed persistent intraoperative equipment and implant errors despite checklist use, suggesting that standard forms may not fully address orthopaedic complexity [5]. Studies should therefore develop and validate speciality-specific SSC modules that include implant verification, instrument readiness, laterality checks, and fluid labelling [10]. Randomised or stepped-wedge trials comparing conventional and orthopaedic-adapted SSCs could help determine whether tailored content translates into measurable reductions in wrong-site surgery, equipment failures, and implant mismatch.

Contextual Determinants and Human Factors

A major knowledge gap lies in understanding the influence of contextual and cultural variables leadership support, team hierarchy, and institutional culture, on SSC efficacy. Studies emphasise that safety interventions succeed only when embedded within a supportive culture that encourages transparency and continuous learning [24]. Research using implementation science frameworks could systematically explore how cultural readiness and leadership engagement mediate checklist success across orthopaedic centres.

Sustainability and Long-Term Outcomes

While the initial implementation of SSCs often improves short-term compliance, longitudinal studies reveal a tendency for “checklist fatigue” and declining adherence over time [1]. Few studies have followed orthopaedic teams beyond 12-18 months post adoption. Future research should evaluate sustainability through prospective cohort designs or registry-based analyses linking SSC use to long-term trends in surgical-site infection, readmission, and reoperation rates. Embedding SSC compliance within institutional quality dashboards may allow continuous feedback and prevent a decline in adherence.

Digitalisation, AI, and Workflow Integration

Although digital checklists are increasingly deployed, their real-world effectiveness is insufficiently validated. Sendlhofer et al. reported that while digital systems improved data capture, they did not guarantee behavioural compliance [25]. Emerging research should assess whether artificial intelligence-assisted SSCs capable of cross-checking EHR entries, imaging data, or implant barcodes enhance clinical reliability without increasing cognitive load. Hybrid implementation studies combining qualitative usability assessment with quantitative outcome analysis would bridge the current gap between technology and human factors [6].

Economic and Workflow Evaluation

Although several studies report the overall cost-effectiveness of SSCs due to complication reduction, orthopaedic-specific economic evaluations are sparse [19]. Orthopaedic surgery’s high implant cost and equipment dependency necessitate rigorous cost-utility analyses comparing SSC-enhanced and conventional workflows. Research integrating Lean and Six Sigma frameworks may clarify how checklists affect theatre efficiency, turnover time, and operative delays.

Standardisation of Outcome Metrics

Heterogeneity in reported outcomes limits meta-analytic synthesis and policy translation. Existing literature uses variable endpoints ranging from infection rates to communication errors, hindering comparison across studies [21]. The development of a Core Outcome Set (COS) for SSC research in orthopaedics, similar to initiatives in general surgery, would enable consistency and allow global benchmarking. Consensus workshops involving surgeons, anaesthetists, and safety scientists should define this framework.

To address these knowledge gaps, future research should focus on developing and validating orthopaedic-specific checklist modules tested through multicentre randomised trials, while employing implementation science methodologies to explore cultural, human-factor, and leadership influences. Establishing longitudinal national registries to track SSC compliance, sustainability, and cost-effectiveness will be crucial to understanding long-term impact. Additionally, evaluating AI-augmented and digital SSC platforms through hybrid effectiveness-implementation trials could help integrate technology into safety practices. Finally, defining and adopting a core outcome set for surgical safety research within orthopaedics would standardise outcome reporting and facilitate global benchmarking. Collectively, these initiatives would shift focus from checklist deployment to optimisation, transforming the SSC from a procedural formality into a dynamic, continuously learning safety system.

## Conclusions

The SSC has become a cornerstone of modern surgical safety, demonstrating measurable reductions in morbidity and mortality across diverse healthcare settings. However, within orthopaedic surgery, its true potential remains underrealised due to procedural complexity, hierarchical theatre culture, and variability in team engagement. Evidence shows that while checklist compliance is often high, genuine behavioural adherence marked by active communication, leadership involvement, and shared accountability is what drives meaningful safety improvement.

For orthopaedics, where implant verification, equipment coordination, and laterality checks are critical, the checklist must evolve beyond a generic template into a speciality-specific, adaptive safety framework. The integration of digital platforms, artificial intelligence, and real-time data auditing offers promising avenues for enhancing accuracy and accountability. Yet, these innovations will succeed only if embedded within a culture that values open dialogue, continuous learning, and mutual respect across professional boundaries. Future progress depends on transforming the SSC from a static procedural tool into a dynamic learning system, one that not only prevents errors but also fosters resilience, reflection, and teamwork. By aligning technical precision with human factors, orthopaedic surgery can lead the next phase of surgical safety innovation, ensuring that every checklist moment represents a genuine safeguard for patient care rather than a perfunctory pause in the operative flow.
